# Plasma levels of soluble ST2, but not IL‐33, correlate with the severity of alcoholic liver disease

**DOI:** 10.1111/jcmm.13990

**Published:** 2018-11-27

**Authors:** Zijian Sun, Binxia Chang, Ang Huang, Shuli Hao, Miaomiao Gao, Ying Sun, Ming Shi, Lei Jin, Wei Zhang, Jun Zhao, Guangju Teng, Lin Han, Hui Tian, Qingsheng Liang, Ji‐Yuan Zhang, Zhengsheng Zou

**Affiliations:** ^1^ Center of Non‐infectious Liver Diseases Peking University 302 Clinical Medical School Beijing China; ^2^ Center of Non‐infectious Liver Diseases Beijing 302 Hospital Beijing China; ^3^ Treatment and Research Center for Infectious Diseases Beijing 302 Hospital Beijing China

**Keywords:** alcoholic liver disease, interleukin‐33, monocyte, sST2

## Abstract

Alcoholic liver disease (ALD) is a complication that is a burden on global health and economy. Interleukin‐33 (IL‐33) is a newly identified member of the IL‐1 cytokine family and is released as an “alarmin” during inflammation. Soluble suppression of tumourigenicity 2 (sST2), an IL‐33 decoy receptor, has been reported as a new biomarker for the severity of systemic and highly inflammatory diseases. Here, we found the levels of plasma sST2, increased with the disease severity from mild to severe ALD. Importantly, the plasma sST2 levels in ALD patients not only correlated with scores for prognostic models (Maddrey's discriminant function, model for end‐stage liver disease and Child‐Pugh scores) and indexes for liver function (total bilirubin, international normalized ratio, albumin, and cholinesterase) but also correlated with neutrophil‐associated factors as well as some proinflammatory cytokines. In vitro, lipopolysaccharide‐activated monocytes down‐regulated transmembrane ST2 receptor but up‐regulated sST2 mRNA and protein expression and produced higher levels of tumour necrosis factor‐α (TNF‐α). By contrast, monocytes pretreated with recombinant sST2 showed decreased TNF‐α production. In addition, although plasma IL‐33 levels were comparable between healthy controls and ALD patients, we found the IL‐33 expression in liver tissues from ALD patients was down‐regulated at both RNA and protein levels. Immunohistochemical staining further showed that the decreased of IL‐33‐positive cells were mainly located in liver lobule area. These results suggested that sST2, but not IL‐33, is closely related to the severity of ALD. Consequently, sST2 could be used as a potential biomarker for predicting the prognosis of ALD.

## 1 INTRODUCTION

Alcoholic liver disease (ALD) is a complex process that covers a broad spectrum of disorders, including simple steatosis, alcoholic hepatitis (AH), alcoholic liver cirrhosis (ALC), and hepatocellular carcinoma (HCC).^1^ Abstinence from drinking is the most effective treatment for patients with ALD, but it is difficult to achieve due to alcohol relapse. Corticosteroids have been used in patients with ALC superimposed severe AH (SAH), but this treatment is only partially effective.^2^ Liver transplantation is a good choice for ALD patients with severe liver dysfunction, as its outcome is better than in patients with other liver diseases.^3^ However, several limitations, such as donors, waiting time, high cost, and multiple complications, restrict the use of liver transplantation.

Alcohol and its metabolite (acetaldehyde)‐induced immune response play an important role in driving the disease progression of ALD. For example, acetaldehyde binds to proteins and DNA to form autoantigens, which could induce mitochondria damage, impair glutathione function, and generate reactive oxygen species. Alcohol abuse also results in colonic microbiota changes and increased intestinal permeability, which leads to elevated serum levels of lipopolysaccharide (LPS), increased LPS levels further trigger innate and adaptive immune responses resulting in proinflammatory cytokine production and pathogenic cell (neutrophils, monocytes) localization to the liver.^4^, ^5^, ^6^, ^7^ More recently, strategies to blockade proinflammatory cytokines have been tested in clinical trials. For instance, an IL‐1R antagonist and an IL‐1 inhibitor are being used in patients with severe AH, and IL‐22 is being used in AH patients.^8^ Identifying the characteristics of novel cytokines in ALD may provide new targets for the diagnosis and treatment of ALD.

Interleukin‐33 (IL‐33) is classified in the IL‐1 cytokine family and is broadly expressed in epithelial cells, endothelial cells, smooth muscle cells, and several organs, such as lung and central nervous system.^9^, ^10^, ^11^ Suppression of tumourigenicity 2 (ST2) is an orphan receptor for IL‐33. There are mainly three expression forms of ST2: a full‐length transmembrane form (ST2L), a soluble form (sST2), and a novel variant.^12^ The IL‐33‐ST2 axis is implicated in the pathogenesis of a variety of conditions, including asthma, rheumatoid arthritis, and Alzheimer's disease.^13^, ^14^, ^15^ In the field of hepatology, the characteristics of the IL‐33‐ST2 axis have been extensively studied. In a diet‐induced nonalcoholic fatty liver disease (NAFLD) mouse model, both the mRNA and protein expression levels of IL‐33 and ST2 were increased in the liver. Treatment with IL‐33 attenuated hepatic steatosis, systemic insulin resistance, and glucose intolerance and reduced serum ALT activity while aggravating hepatic fibrosis. Furthermore, IL‐33 can promote the type 2 T helper cell (Th2) response and M2 macrophage activation and beneficially modulate the expression profiles of fatty acid metabolism genes in the liver. IL‐33 did not affect hepatic steatosis and fibrosis in a ST2^−/−^ NAFLD mice model. Moreover, in the liver of NAFLD patients, IL‐33 and ST2 mRNA expression levels were increased with the progression of the disease. To sum up, treatment with IL‐33 attenuated hepatic steatosis induced by diet but aggravated hepatic fibrosis through an ST2‐dependent manner.^16^ In patients with hepatitis B virus infection or hepatitis C virus infection, plasma IL‐33 levels were significantly increased, and the concentration changes were consistent with serum ALT levels.^17^, ^18^ The protective role of the IL‐33‐ST2 axis in concanavalin A‐induced severe hepatitis has received attention. After ST2‐/‐ mice were treated with concanavalin A, they exhibited a high number of mononuclear cells in the liver and high levels of proinflammatory tumour necrosis factor (TNF)‐α and interferon (IFN)‐γ. However, the numbers of CD4^+^ Foxp3^+^ cells were significantly higher in WT mice. Furthermore, WT mice treated with IL‐33 had attenuated liver damage and increased CD4^+^ Foxp3^+^ cells in the liver. IL‐33 also suppressed caspase‐3 activation and BAX expression and enhanced Bcl‐2 expression in the liver.^19^ When NKT‐deficient mice or TNF‐related apoptosis‐inducing ligand (TRAIL)‐deficient mice received concanavalin A treatment, IL‐33 expression in hepatocytes was also inhibited. These results suggested that NKT cells and TRAIL are responsible for inducing IL‐33 during acute hepatitis.^20^, ^21^ Many studies have shown that the IL‐33‐ST2 axis is associated with liver fibrosis. IL‐33 and ST2 were overexpressed at the mRNA level in mouse and human fibrotic livers, and IL‐33 expression was correlated with collagen expression.^22^ Mechanism investigations revealed that innate lymphoid cell type 2 (ILC2) was involved in the process. IL‐33 led to the activation and accumulation of ILC2 through ST2 signalling in the liver. IL‐33‐mediated ILC2 produced IL‐13, and IL‐13 then initiated the activation and differentiation of hepatic stellate cells (HSCs) via the IL‐4Rα‐STAT6 transcription factor‐dependent pathway. In addition, in ST2‐deficient liver fibrosis mice, HSC activation was decreased, and IL‐33 could activate HSCs in vitro.^23^, ^24^ Recently, Wang and coworkers found a dual role of the IL‐33‐ST2 axis during ALD development. In mice with mild ALD, ST2 dampened the inflammatory activation of hepatic macrophages through inhibiting the NF‐κB pathway and played a protective role in an IL‐33‐independent  manner. In contrast, at the later and severe stages of ALD, extracellular IL‐33 was markedly increased, thus triggering IL‐33‐ST2 signalling and resulting in significant cell death and liver injury.^25^

In the present study, we further assessed the characteristics of the IL‐33‐ST2 axis in a cohort of patients with ALD and found that serum sST2 levels were profoundly increased in patients with ALD and were closely associated with disease progression.

## 2 MATERIALS AND METHODS

### 2.1 Patients

Forty‐six patients with ALD were enrolled in the study who have an excess alcohol consumption history (>30 g/d) together with the indication of liver injury such as clinical and/or biological abnormalities and were diagnosed according to existing criteria,^26^ including eight mild ALD patients without liver cirrhosis (MALD) all confirmed by liver biopsy and 38 patients progressing to the stage of ALC diagnosed using ultrasonography, magnetic resonance imaging or computed tomography techniques. Individuals with concurrent HBV infection, HCV infection, autoimmune liver diseases, and severe systemic diseases were excluded. Of the ALC patients, a subset was divided further into 15 patients with ALC superimposed SAH (ALC+SAH) based on Maddrey's discriminant function (MDF) scores of more than 32. In conclusion, patients with ALD were divided into three groups, that is, MALD group without liver cirrhosis, ALC group, and ALC+SAH group. The MDF formula was 4.6 × [patient prothrombin time (PT) (s) − matched control PT (s)] + [serum bilirubin (μmol/L)/17.1)].^27^, ^28^ The model for end‐stage liver disease (MELD) and Child‐Pugh scores were also calculated.

The study was approved by the Beijing 302 Hospital Ethics Committee, and written informed consent was obtained from the participants or their relatives. The baseline clinical data of all patients and healthy controls (HCs) are summarized in Table 1.

**Table 1:** Clinical characteristics of study subjects

Groups	HC (n = 20)	MALD (n = 8)	ALC (n = 23)	ALC+SAH (n = 15)
Age, years	29 (21‐38)	47 (35‐67)	52 (34‐64)	44 (32‐63)
Male, n (%)	20 (100)	8 (100)	23 (100)	15 (100)
WBC, 10^9^/L	6.5 (4.8‐8.1)	5.4 (4.5‐10.4)	3.6 (1.1‐19.9)	7.8 (1.9‐29.1)
NEU#, 10^9^/L	3.9 (2.1‐4.9)	2.5 (1.9‐6.1)	2.0 (0.8‐14.7)	4.7 (1.3‐16.2)
LYM#, 10^9^/L	2.2 (1.6‐2.9)	2.3 (1.5‐3.6)	1.1 (0.1‐3.2)	1.2 (0.5‐4.1)
NLR	1.8 (1.0‐2.5)	1.3 (0.7‐3.3)	2.0 (0.5‐11.6)	3.4 (2.1‐11.6)
HGB, g/L	155 (128‐167)	148 (109‐168)	109 (54‐154)	86 (55‐119)
PLT, 10^9^/L	235 (184‐314)	213 (174‐250)	76 (24‐281)	59 (19‐245)
PT, s	—	10.8 (10.6‐12.7)	14.3 (10.3‐18.5)	20.3 (17.0‐25.4)
INR	—	0.9 (0.9‐1.1)	1.3 (0.9‐1.6)	1.8 (1.5‐2.3)
ALT, U/L	26 (14‐66)	163 (64‐447)	27 (9‐55)	30 (13‐48)
AST, U/L	23 (17‐63)	144 (23‐637)	43 (20‐154)	55 (25‐118)
AST/ALT	1.0 (0.7‐1.4)	0.9 (0.4‐1.5)	1.7 (0.9‐3.4)	2.2 (1.7‐3.0)
TBIL, μmol/L	13.1 (8.1‐23.3)	11.1 (5.9‐78.2)	28.8 (5.3‐98.0)	173.9 (92.1‐331.6)
ALB, g/L	47 (44‐50)	40 (31‐45)	32 (21‐42)	31 (22‐37)
CHE, U/L	—	7067 (4230‐10688)	3324 (1210‐6250)	1762 (755‐3527)
ALP, U/L	—	141 (57‐220)	122 (66‐262)	147 (77‐280)
GGT, U/L	—	179 (19‐2253)	65 (11‐666)	46 (16‐321)
CR, μmol/L	—	69 (52‐97)	62 (36‐343)	82 (53‐149)
MDF score	—	−6.3 (−7.2 to 4.6)	11.3 (−8.2 to 31.4)	44.7 (34.1‐74.3)
MELD score	—	−4.5 (−8.7 to 3.4)	2.9 (−6.9 to 22.3)	14.7 (10.9‐19.9)
Child‐Pugh score	—	5 (5‐8)	7 (5‐11)	11 (8‐13)

HC, healthy control; MALD, mild alcoholic liver disease; ALC, alcoholic liver cirrhosis; ALC+SAH, alcoholic liver cirrhosis superimposed severe alcoholic hepatitis; WBC, white blood cell; NEU#, absolute value of neutrophils; LYM#, absolute value of lymphocytes; NLR, neutrophil‐to‐lymphocyte ratio; HGB, haemoglobin; PLT, platelet; PT, prothrombin time; INR, international normalized ratio; ALT, alanine aminotransferase; AST, aspartate transaminase; TBIL, total bilirubin; ALB, albumin; CHE, cholinesterase; ALP, alkaline phosphatase; GGT, gamma‐glutamyl transferase; CR, creatinine; MDF, Maddrey's discriminant function; MELD, model for end‐stage liver disease.

Data are shown as number (%) or median (range).

Liver biopsies from five MALD patients undergoing diagnosis, liver tissues from four ALC patients undergoing liver transplantation, and nine healthy liver donors were obtained for subsequent studies.

### 2.2 Microarray assay

Total RNA was extracted from the liver biopsy tissues using a QIAGEN RNeasy^®^ Mini Kit (Qiagen, Hilden, Germany) and then quantified with a NanoDrop ND‐2000 spectrophotometer (Thermo Scientific, Waltham, MA, USA). The RNA integrity was further assessed using an Agilent Bioanalyzer 2100 (Agilent Technologies, Santa Clara, CA, USA). Total RNA was transcribed into double‐stranded cDNA and then hybridized onto an Affymetrix PrimeView Human Gene Expression Array and scanned with an Affymetrix Scanner 3000 (Affymetrix, Thermo Scientific). Raw data were obtained with an Affymetrix GeneChip Command Console (version 4.0, Affymetrix, Thermo Scientific) and subjected to a basic analysis using Genespring software (version 13.1, Agilent Technologies).

### 2.3 Quantitative real‐time PCR (qPCR)

mRNA was extracted using TRIzol reagent (Thermo Scientific). First‐strand cDNA was synthesized using a RevertAid First Strand cDNA Synthesis Kit (Thermo Scientific), and qPCR amplification was performed using SYBR Green Master Mix (Applied Biosystems, CA, USA) according to the manufacturer's instructions. Primers used for the target genes were as follows: IL‐33 (forward primer, 5′ ‐ GGAAGAACACAGCAAGCAAAGCCT ‐ 3′; reverse primer, 5′ ‐ TAAGGCCAGAGCGGAGCTTCATAA ‐ 3′), ST2L (forward primer, 5′ ‐ AGGCTTTTCTCTGTTTCCAGTAATCGG ‐ 3′; reverse primer, 5′ ‐ GGCCTCAATCCAGAACATTTTTAGGATGATAAC ‐ 3′), and sST2 (forward primer, 5′ ‐ GAAGGCACACCGTAAGACTA ‐ 3′; reverse primer, 5′ ‐ GACAAACCAACGATAGGAGG ‐ 3′). β‐Actin was used as an endogenous control (forward primer, 5′ ‐ CATGTACGTTGCTATCCAGGC ‐ 3′; reverse primer, 5′‐ CTCCTTAATGTCACGCACGAT ‐ 3′). These primers were synthesized by Invitrogen (Beijing, China). The specificity of the amplification was determined by melting curve analysis. The relative fold change in each gene was calculated using the 2(−ΔΔC(T)) method.^29^

### 2.4 Western blot analysis

Proteins were extracted from the liver tissues, and the concentrations were determined using the Bradford method. A total of 30 μg total soluble proteins was separated by 12% sodium dodecyl sulphate‐polyacrylamide gel electrophoresis and transferred onto a polyvinyl difluoride membrane. The membrane was blocked and incubated with a primary anti‐IL‐33 antibody (1:500, ab118503, Abcam, Cambridge, UK) and anti‐β‐actin antibody (1:2000, 4967, Cell Signaling Technology, Boston, MA, USA) for 3 h at room temperature. The membrane was then incubated with a secondary antibody (goat anti‐rabbit IgG peroxidase conjugate, 1:3000, Abcam) and further detected using an enhanced chemiluminescence detection kit (Engreen Biosystem, Beijing, China). ImageJ was used for greyscale analysis, and β‐actin was employed as the loading control.

### 2.5 Immunohistochemical staining

Formalin‐fixed, paraffin‐embedded liver tissue sections were incubated with an anti‐IL‐33 antibody overnight at 4°C after boiling in an ethylenediaminetetraacetic acid antigen retrieval solution (pH 8.0) at 98°C for 10 min for antigen‐epitope retrieval and blocking endogenous peroxidase activity with 0.3% hydrogen peroxide. Then, the sections were incubated for 30 minutes with poly HRP‐anti‐Mo/Rb IgG secondary antibody (Zsbio, Beijing, China) at 37°C, and staining was performed using 3‐amino‐9‐ethylcarbazole (AEC, red colour) as a colorimetric substrate, followed by counterstaining with haematoxylin. The IL‐33 expression levels for the semiquantitative assay were measured with Image‐Pro Plus 6.0 software (Media Cybernetics, CA, USA) and expressed as the integrated optical density (IOD) parameter.

### 2.6 Monocyte isolation and activation

CD14^+^ monocytes were separated using a Pan Monocyte Isolation Kit (Miltenyi Biotec, Bergisch Gladbach, Germany) according to the manufacturer's instructions. The purity of the separated monocytes was >90%. The purified monocytes were activated using LPS (1000 ng/mL, Sigma, St. Louis, USA) or an equal volume of normal saline for 6 h. After stimulation, the supernatant from the culture was used for sST2 detection, and adherent monocytes were collected for surface ST2 detection and message ST2 detection.

For investigating the effect of sST2 on monocytes, purified monocytes were pretreated with or without recombinant sST2 (rsST2, 100 ng/mL, R&D Systems, Minneapolis, MN, USA) for 1 h and were further stimulated with 1000 ng/mL LPS or an equal volume of normal saline for 6 h. Subsequently, the levels of TNF‐α production in monocytes were detected by flow cytometry.

### 2.7 Flow cytometric analysis

For extracellular staining, isolated and cultured monocytes were first blocked with FcR block (Human TruStain FcX, Biolegend, San Diego, CA, USA) at 37°C for 30 min. Then, the cells were stained with antibodies for anti‐CD14 (325606, Biolegend), anti‐ST2L, or anti‐IgG1 isotype control (101002F and 1053002F, MD Biosciences, Oakdale, MN, USA) and incubated for 20 min at 4°C in the dark. For intracellular TNF‐α staining, monocytes were permeabilized, fixed, and stained using the Fixation/Permeabilization Kit according to manufacturer's instructions (BD Biosciences, San Jose, CA, USA) then were stained with antibody specific to TNF‐α (502912, Biolegend) as described above. The stained monocytes were analysed with a FACS Calibur flow cytometer (BD Biosciences) and FlowJo software (TreeStar, Ashland, OR, USA).

### 2.8 Enzyme‐linked immunosorbent assay (ELISA)

Commercial ELISA kits were used to measure IL‐33, IL‐1β, IL‐6 (BioLegend), soluble cluster of differentiation 14 (sCD14), intestinal fatty acid binding protein (I‐FABP), and sST2 (R&D Systems) according to the manufacturers’ protocols.

### 2.9 Statistical analysis

Continuous variables are expressed as the median with the range or the mean ± standard deviation (SD). Comparisons between various individuals were performed with the Mann‐Whitney U test, whereas multiple comparisons between different groups were analysed using the Kruskal‐Wallis H nonparametric test. The Wilcoxon matched‐pair *t* test was applied to comparisons between the same individual after different treatments. Correlations between variables were evaluated by Spearman's rank correlation test. All statistics were performed using SPSS20.0, and a two‐sided *P*‐value <0.05 indicated statistical significance.

## 3 RESULTS

### 3.1 Increased plasma levels of sST2, but not IL‐33, were associated with ALD severity

**Figure 1:**
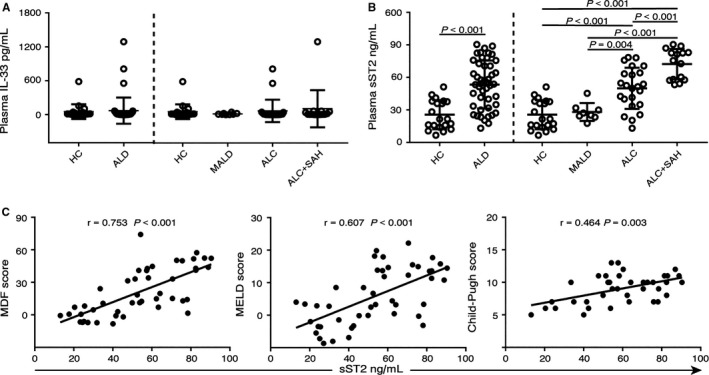
Plasma levels of sST2 increased with the disease severity in ALD patients. (A‐B) Plasma levels of IL‐33 and sST2 in 20 HCs and 46 ALD patients, including 8 MALD and 38 ALC patients (including 15 patients with ALC+SAH). (C) Correlation analysis of plasma sST2 levels and prognosis scores (MDF scores and MELD scores were calculated for all ALD patients, and Child‐Pugh scores were calculated for ALC patients)

We measured plasma IL‐33 and sST2 levels in a cohort of patients with ALD and HCs. We found that the plasma levels of IL‐33 were similar between the HCs and all of the patients with ALD, and no differences were observed between each group (Figure 1A).

We then detected the plasma levels of sST2, which were significantly higher in patients with ALD (median 54.0 ng/mL, range 13.1‐90.5 ng/mL) than in the HCs (median 21.6 ng/mL, range 6.6‐51.3 ng/mL, *P* < 0.001). However, the plasma sST2 concentrations were not different between the MALD group (MALD: median 26.2 ng/mL, range 17.6‐45.4 ng/mL) and the HC group. Interestingly, the plasma concentrations of sST2 were significantly higher in patients with ALC (median 51.4 ng/mL, range 13.1‐78.5 ng/mL) than in HCs and patients with MALD. Particularly in ALC+SAH patients, the plasma sST2 concentrations (median 75.6 ng/mL, range 53.3‐90.5 ng/mL) were further increased compared to those in ALC patients without severe liver damage (HC vs ALC:

*P* < 0.001, HC vs ALC+SAH: *P* < 0.001, MALD vs ALC: *P* = 0.004, MALD vs ALC+SAH: *P* < 0.001, ALC vs ALC+SAH: *P* < 0.001, Figure 1B).

We analysed the correlations between plasma sST2 concentrations and the prognostic model scores in the ALD patients. Plasma sST2 levels correlated positively with both MDF scores (*r*  = 0.753, *P* < 0.001) and with MELD scores (*r*  = 0.607, *P* < 0.001). Increased sST2 levels were also correlated positively with the Child‐Pugh score (*r*  =  0.464, *P* = 0.003, Figure 1C) within ALC patients (ALC and ALC+SAH groups). These data suggested that the increased plasma levels of sST2 were associated with the ALD severity.

### 3.2 Assessment of the potential correlations between sST2 levels and liver damage markers, liver proteosynthetic and reserve function, and neutrophil‐associated indexes

**Figure 2:**
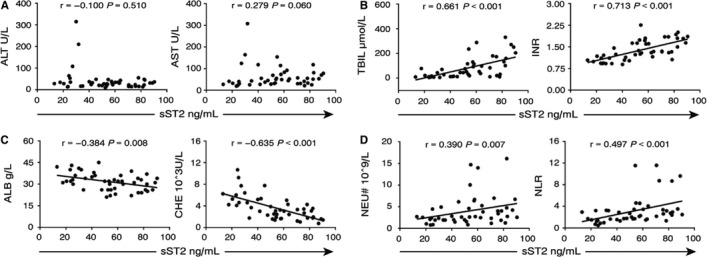
Correlation analysis of plasma sST2 levels and clinical indexes in ALD patients. (A‐B) Correlation analysis of plasma sST2 levels and liver damage parameters (ALT, AST, and TBIL levels and the INR) in 46 ALD patients. (C) Correlation analysis of plasma sST2 levels and liver proteosynthetic and reserve function markers (ALB and CHE) in 46 ALD patients. (D) Correlation analysis of plasma sST2 levels and neutrophil‐associated indexes (NEU# and NLR) in 46 ALD patients

Alanine aminotransferase (ALT) and aspartate transaminase (AST) are important indicators of early liver damage. However, we did not observe any correlation between plasma sST2 levels and ALT or AST levels (Figure 2A). By contrast, sST2 levels were positively correlated with serum total bilirubin levels (TBIL, *r* = 0.661, *P* < 0.001) and the international normalized ratio (INR, *r *  =  0.713, *P* < 0.001, Figure 2B), both of which are markers for later liver damage.

Albumin (ALB) and cholinesterase (CHE) were used to reflect the liver proteosynthetic and reserve function. We observed inverse correlations between plasma sST2 and serum ALB (*r*  =  −0.384, *P* = 0.008) and CHE (*r*   =  −0.635, *P* < 0.001, Figure 2C) levels.

Neutrophils are considered to be the primary cell type responsible for liver damage in ALD, and the neutrophil‐to‐lymphocyte ratio (NLR) was recently identified to be associated with adverse prognosis in some diseases.^30^ We found positive correlations between sST2 levels and the absolute value of neutrophils (NEU#, *r* = 0.390, *P* = 0.007) and the NLR (*r* = 0.497, *P* < 0.001, Figure 2D). These results suggest a link between sST2 levels and the immune response involving neutrophils.

### 3.3 Assessment of the potential correlations between sST2 levels and markers for inflammation and gut damage

**Figure 3:**
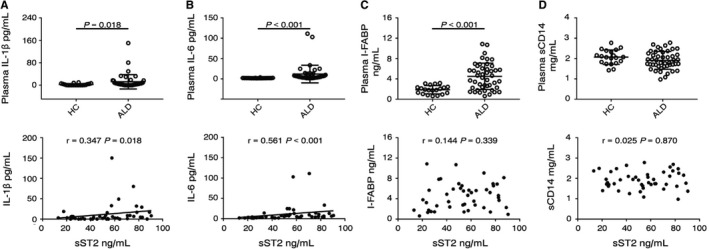
Assessment of potential correlations between sST2 levels and inflammation markers. (A‐D) Plasma levels of IL‐1β, IL‐6, I‐FABP, and sCD14 in 20 HCs and 46 ALD patients and correlation analyses of plasma sST2 and the above molecules in 46 ALD patients

Inflammatory cytokines, including IL‐1β and IL‐6, are important pathogenic factors that contribute to the development of ALD. In agreement with the literature, serum levels of both IL‐1β and IL‐6 were significantly increased in ALD patients (IL‐1β HC: median 0.4 pg/mL, range 0.0‐9.9 pg/mL, ALD: median 3.9 pg/mL, range 0.0‐150.3 pg/mL, HC vs ALD: *P* = 0.018; IL‐6 HC: median 1.9 pg/mL, range 1.5‐3.6 pg/mL, ALD: median 5.2 pg/mL, range 1.7‐111.3 pg/mL, HC vs ALD: *P* < 0.001, Figure 3A and B). We observed that sST2 levels were positively correlated with IL‐1β and IL‐6 levels (IL‐1β, *r* = 0.347, *P* = 0.018; IL‐6, *r* = 0.561, *P* < 0.001).

Intestinal fatty acid binding protein (I‐FABP) is expressed in epithelial cells and released when intestinal mucosal damage occurs.^31^ We found that serum I‐FABP levels were significantly increased in ALD patients (HC: median 1.9 ng/mL, range 0.6‐3.2 ng/mL, ALD: median 4.4 ng/mL, range 0.6‐10.9 ng/mL, HC vs ALD: *P* < 0.001, Figure 3C). However, no correlation was observed between plasma sST2 levels and I‐FABP.

Serum sCD14 levels can be used as a marker for LPS bioactivity in HIV infection.^32^ However, the plasma levels of sCD14 were not different between HCs and ALD patients, and no correlation was observed between plasma sST2 and sCD14 levels (Figure 3D).

### 3.4 LPS‐activated monocytes were one of the major cell types involved in sST2 production

**Figure 4:**
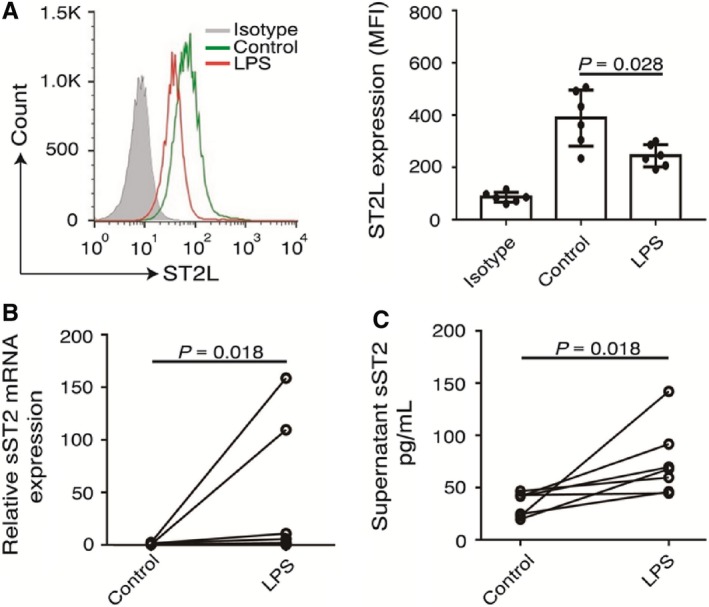
Profile of ST2 expression in LPS‐activated monocytes. (A) Flow cytometric analysis of ST2L expression changes in monocytes stimulated with 1000 ng/mL LPS for 6 hours in vitro (n = 6 per group). IgG1 was used as an isotype control, and MFI values for ST2L are shown. (B‐C) qPCR analysis of the changes in sST2 mRNA expression in monocytes and sST2 concentrations in the supernatants after LPS treatment as in (A) (n = 7 per group)

ST2L is expressed mainly on various haematopoietic cells, including Th2 cells, mast cells, and eosinophils.^33^, ^34^ However, the cell types associated with sST2 are unclear. We further investigated whether monocytes were associated with ST2. Transmembrane receptor ST2L expression was significantly lower in the LPS‐activated monocyte group (MFI: 389 ± 107.7) than in the control group without LPS stimulation (MFI: 244 ± 42.4, *P* = 0.028, Figure 4A). Interestingly, there was no significant difference in the ST2L mRNA levels of monocytes with or without LPS stimulation. By contrast, the mRNA levels of sST2 were significantly increased after the monocytes were treated with LPS (control group: 1.00 ± 0.81; LPS group: 41.24 ± 65.23, *P* = 0.018, Figure 4B). Furthermore, the sST2 concentrations in the supernatants from the LPS group (74.63 ± 33.82 pg/mL) were markedly higher than those in the paired control group (34.37 ± 11.30 pg/mL, *P* = 0.018, Figure 4C).

### 3.5 sST2 attenuated TNF‐α production from LPS‐activated monocytes

**Figure 5:**
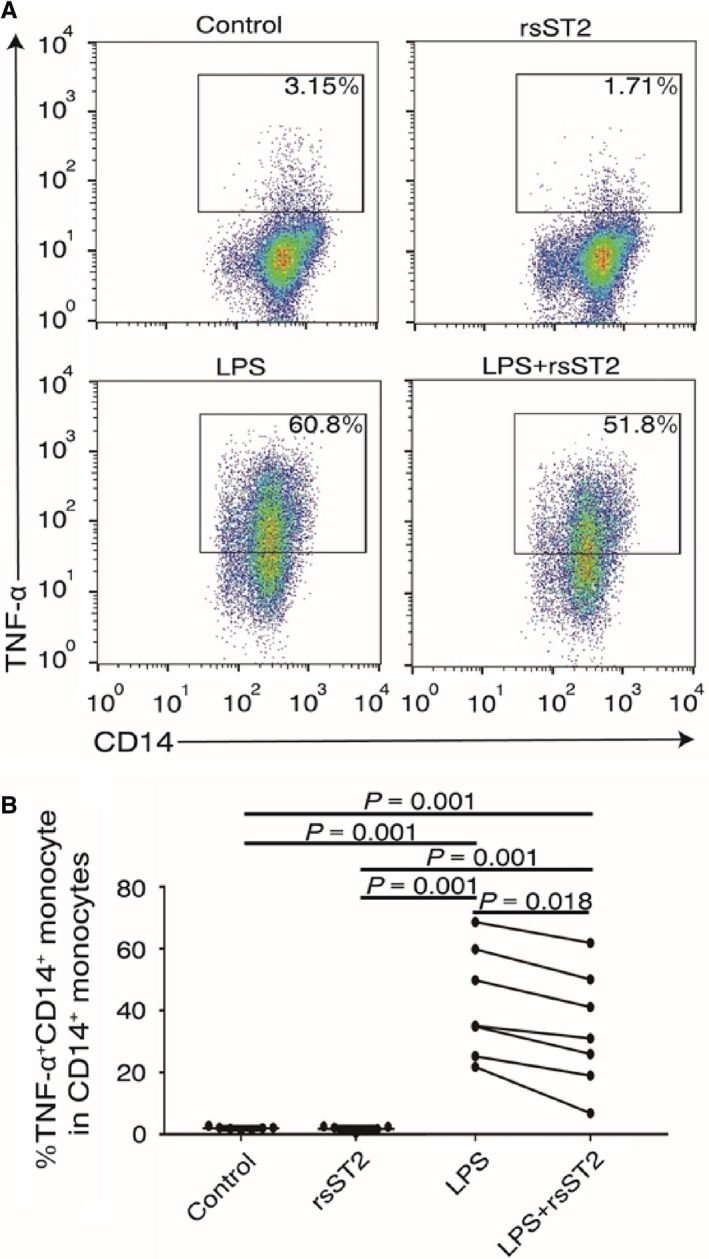
sST2 attenuated TNF‐α production from LPS‐activated monocytes. (A) Flow cytometric analysis of TNF‐α production from monocytes stimulated with 1000 ng/mL LPS for 6 hours in vitro with or without 1 hour rsST2 pretreatment (100 ng/ml, n = 7 per group). (B) Pooled data showing the TNF‐α expressing monocytes in response to rsST2 pretreatment under stimulation with LPS

TNF‐α has been reported to be one of representative proinflammatory cytokines produced through NF‐κB pathway in monocytes.^35^ To detect the effect of sST2 on monocyte responses, monocytes isolated from patients with ALD were stimulated with LPS in the presence or absence of rsST2. As showed in Figure 5, monocytes produced higher levels of TNF‐α after stimulated by LPS, suggested LPS effectively activates NF‐κB pathway in vitro (TNF‐α^+^ CD14^+^%, control group: 1.89 ± 0.50; LPS group: 41.50 ± 17.44, *P* = 0.001). Of note, rsST2 itself has no effect on inducing TNF‐α production from monocytes. Importantly, we found that pre‐rsST2‐treated monocytes significantly down‐regulate TNF‐α production in response to LPS, suggested sST2 partially attenuated the response of LPS‐activated monocytes (LPS+rsST2 group: 33.14 ± 18.54, *P* = 0.018).

### 3.6 IL‐33 expression was decreased in the livers of ALD patients

**Figure 6:**
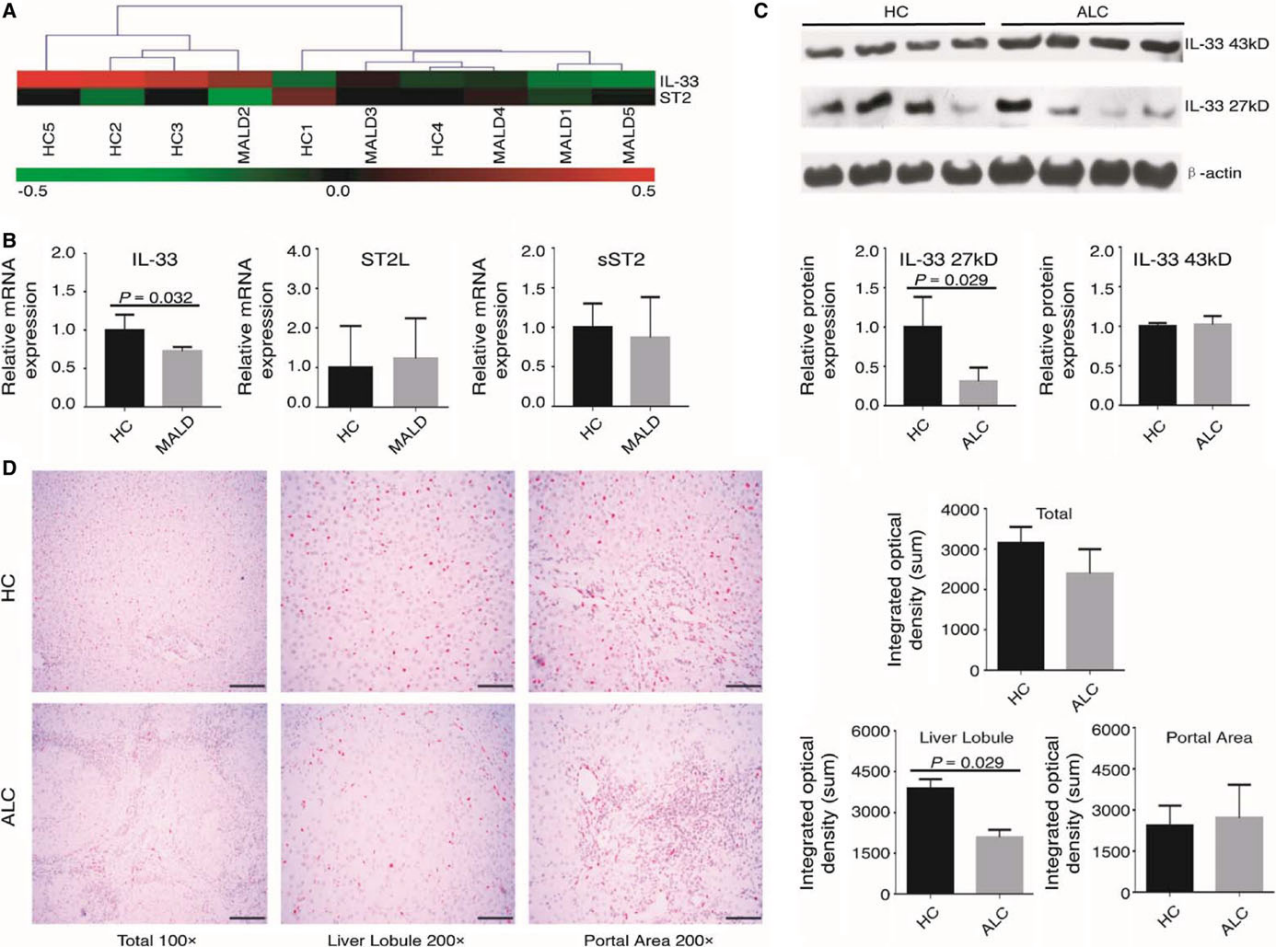
Decreased IL‐33 expression in the liver tissues of ALD patients. (A) Heatmap of IL‐33 and ST2 gene expression in the livers of HCs (n = 5) and MALD patients (n = 5). (B) qPCR analysis of IL‐33 and ST2 (including ST2L and sST2) mRNA expression in the same liver samples as above. (C) Western blot analysis of IL‐33 protein expression in the livers of HCs (n = 4) and ALC patients (n = 4). β‐actin was used as a loading control, and the densitometry results are shown. (D) Immunohistochemical staining for IL‐33 in the total view (100 ×  magnification), liver lobule (200 ×  magnification), and portal area (200 ×  magnification) of livers from HCs (n = 4) and ALC patients (n = 4). IOD values for IL‐33 expression are shown

We further examined whether there was a difference in IL‐33 expression in the livers of ALD patients and HCs. The heatmap in Figure 6A shows that IL‐33 gene expression slightly decreased in MALD group, whereas no difference was observed in the ST2 levels. IL‐33 and ST2 gene expression was further validated by qPCR. In agreement with the microarray data, IL‐33 mRNA levels were lower in the livers of MALD patients than in those of the HCs (HCs: 1.00 ± 0.20; MALD: 0.72 ± 0.06, *P* = 0.032, Figure 6B), whereas ST2L and sST2 mRNA levels were similar between the two groups.

We then evaluated IL‐33 protein levels by Western blot. As shown by the densitometry results in Figure 6C, 27 kD IL‐33 levels, but not 43 kD IL‐33 levels, were significantly down‐regulated in ALC patients (HCs: 1.00 ± 0.38; ALC: 0.31 ± 0.18, *P* = 0.029).

We also detected the distribution of IL‐33‐positive cells in the livers of ALC patients and HCs. We found that IL‐33 protein was stained negatively in hepatocytes, while the detectable IL‐33^+^ cells were located in the inflammatory portal areas and in hepatic sinusoids in the lobule areas. As shown in Figure 6D, the relative levels of IL‐33 were lower in ALC patients than in HCs (IOD of HCs: 3880 ± 341.0; IOD of ALC: 2086 ± 280.8, *P* = 0.029). Interestingly, the relative levels of IL‐33 in the lobule area, but not in the portal area, were decreased significantly in the livers of ALC patients.

## 4 DISCUSSION

The IL‐33‐ST2 axis has been reported to be involved in a variety of liver diseases; recently, an unanticipated role of this axis has been demonstrated in a mouse model of ALD.^25^ In the present study, we further investigated the characteristics of the IL‐33‐ST2 axis in a cohort of patients with ALD. We found that serum sST2, but not IL‐33, levels were increased in patients with ALD. Thus, serum sST2 levels could be a promising biomarker in predicting the clinical outcomes of ALD.

Previous studies have reported that sST2 can be used as a prognostic biomarker for the development of systemic and highly inflammatory diseases, such as aortic dissection, sepsis, and heart failure as well as mortality in patients with these diseases.^36^, ^37^, ^38^ This hypothesis is reinforced by the finding that plasma sST2 concentrations could predict poor prognosis in patients with acute‐on‐chronic hepatitis B liver failure.^39^ The present study further revealed its prognostic value in predicting clinical outcomes in patients with ALD. There are three lines of evidence to support this finding. First, plasma sST2 concentrations were not increased in patients with MALD, whereas they were increased significantly in patients with ALC. Second, ALC patients with SAH, who often present with clinically exacerbated episodes following certain precipitating events and provide a compatible control for ALC patients with mild hepatitis, exhibited greater increases in plasma sST2 concentrations than ALC patients with mild hepatitis. In addition, in line with the data of Lei et al showing increased serum concentrations of sST2 in patients with acute‐on‐chronic hepatitis B liver failure,^39^ we also observed that sST2 positively correlated with TBIL levels and the INR, both of which are primary factors for evaluating liver failure.^40^ These data indicated that sST2 correlated with the disease progression of ALD. Third, sST2 levels showed a strong positive correlation with MDF scores; scores greater than 32 indicated that the ALD patient was in a severe, life‐threatening condition according to this scoring system.^41^ In addition, sST2 positively correlated with MELD scores; the higher this score was, the more urgent the need for liver transplantation was.^42^ What is more, sST2 also positively correlated with Child‐Pugh scores; this is the most commonly used scoring system for evaluating the prognosis of liver cirrhosis.^43^ Taken together, these observations indicate that plasma sST2 concentrations in ALD patients have prognostic value in predicting clinical outcomes.

Importantly, our data also revealed a clear correlation between plasma sST2 concentrations and proinflammatory markers. In this regard, neutrophils are one of the key pathogenic cell types in ALD.^44^ We found that sST2 positively correlated with the neutrophil number and NLR. This association was further supported by the data that sST2 positively correlated with the proinflammatory cytokines IL‐1β and IL‐6. This observation is clinically relevant as both IL‐1β and IL‐6 plasma levels are involved in inducing liver injury in patients with ALD.^45^ Thus, the plasma sST2 level is a good indicator of inflammatory levels in ALD patients.

Previous studies suggested that IL‐33 exerts anti‐inflammatory properties in models of heart disease, obesity, and uveitis. The protective properties of IL‐33 are associated with increased circulating Th2 cytokine levels, decreased IFN‐γ production, increased eosinophil numbers, and type 2 innate lymphoid cells.^46^ More recently, IL‐33 has also been shown to mediate immunosuppression and tissue repair by activating regulatory T cells (Treg) and promoting M2 macrophage polarization.^47^ In the present study, we investigated the characteristics of IL‐33 in a cohort of ALD patients and found that the levels of IL‐33 in liver tissues were decreased in patients with MALD and ALC. This finding was in line with the available observation that the liver IL‐33 concentration was decreased in HBV‐ACLF patients with accompanying liver cirrhosis.^39^ Thus, we may speculate that by interfering with IL‐33, the increased sST2 levels further block bioactivity with respect to the function of IL‐33. Liver cirrhosis is the consequence of progressive fibrosis; the present study, in contrast to a recent report of IL‐33‐ST2 axis characteristics in a mouse model of AH and fibrosis,^25^ further extends the characteristics of the IL‐33‐ST2 axis in this type of patients.

The detailed bioactivity of sST2 remains unknown in ALD patients. Clinically, ALC is usually accompanied by portal hypertension and increased gut permeability, both of which contribute to the accumulation of pathogen‐associated molecules, such as LPS, in the blood circulation.^48^ In the present study, we found that serum I‐FABP levels, a marker for gut permeability, were significantly increased in ALD patients. Furthermore, LPS‐activated monocytes produced sST2 as well as some proinflammatory cytokines, such as TNF‐α, which has been proved to be involved in the pathogenesis of ALD.^49^ It has been found that both serum and liver levels of TNF‐α increased in ALD patients.^50^ Our experiments have shown that sST2 administration could attenuate an LPS challenge and down‐regulate the inflammatory or activation processes, evident by the down‐regulation of LPS‐induced TNF‐α production in monocytes. Therefore, we hypothesized that the sST2 production of LPS‐activated monocytes may be a protective feedback mechanism responding to inflammatory stress. Notably, a previous study found that vein and arterial endothelial cells, as well as microvascular endothelial cells, express sST2 under stress and hypertension conditions.^51^ Future studies should investigate whether portal hypertension is related to the increased plasma sST2 levels in ALD patients.

Taken together, our data show that high plasma levels of sST2 are associated with prognostic scores for disease progression and correlate well with biomarkers for inflammation. These results, for the first time, demonstrate the characteristics of the IL‐33‐ST2 axis in a cohort of patients with ALD, including liver cirrhosis with severe hepatitis. Our findings suggest that levels of serum sST2, but not IL‐33, are closely related to the severity of ALD and could be used as a potential biomarker for predicting the prognosis of ALD.

## CONFLICT OF INTEREST

The authors declare no conflict of interest.
